# A Portable Quantum Cascade Laser Spectrometer for Atmospheric Measurements of Carbon Monoxide

**DOI:** 10.3390/s18072380

**Published:** 2018-07-21

**Authors:** Silvia Viciani, Alessio Montori, Antonio Chiarugi, Francesco D’Amato

**Affiliations:** 1 National Research Council—National Institute of Optics (CNR-INO), Largo E. Fermi 6, 50125 Firenze, Italy; silvia.viciani@ino.cnr.it; 2 National Research Council—National Institute of Optics (CNR-INO), Via N. Carrara 1, 50019 Sesto Fiorentino, Italy; alessio.montori@ino.cnr.it; 3 National Institute of Geophysics and Vulcanology (INGV), Sez. di Pisa, Via della Faggiola 32, 56126 Pisa, Italy; antonio.chiarugi@ingv.it

**Keywords:** in situ sensing, airborne measurements, stratosphere, carbon monoxide, climate change

## Abstract

Trace gas concentration measurements in the stratosphere and troposphere are critically required as inputs to constrain climate models. For this purpose, measurement campaigns on stratospheric aircraft and balloons are being carried out all over the world, each one involving sensors which are tailored for the specific gas and environmental conditions. This paper describes an automated, portable, mid-infrared quantum cascade laser spectrometer, for in situ carbon monoxide mixing ratio measurements in the stratosphere and troposphere. The instrument was designed to be versatile, suitable for easy installation on different platforms and capable of operating completely unattended, without the presence of an operator, not only during one flight but for the whole period of a campaign. The spectrometer features a small size (80 × 25 × 41 cm${}^{3}$), light weight (23 kg) and low power consumption (85 W typical), without being pressurized and without the need of calibration on the ground or during in-flight operation. The device was tested in the laboratory and in-field during a research campaign carried out in Nepal in summer 2017, onboard the stratospheric aircraft M55 Geophysica. The instrument worked extremely well, without external maintenance during all flights, proving an in-flight sensitivity of 1–2 ppbV with a time resolution of 1 s.

## 1. Introduction

A detailed knowledge of composition and evolution of the atmosphere is fundamental for the inputs to any predictive climate model. The chemistry of atmospheric compounds, their transportation around the world and their evolution under climate changes provide essential information for any climatological study and forecast. Some gases play the role of tracers, as they exhibit particular features in terms of sources, reactivity, concentration and lifetime. Carbon Monoxide (CO) is an ideal tracer of atmospheric transport for several reasons. Its mixing ratio decreases of about one order of magnitude when moving from the troposphere, where its lifetime does not exceed a couple of months, to the stratosphere, where it can reside up to several months at lower concentrations. So, CO can be used as tracer of air transport across the tropopause, witnessed by too low mixing ratio in the troposphere or too high mixing ratio in the stratosphere, as might occur during strong convective activity [1]. As one of the CO sources in the troposphere is combustion [2,3], CO is also an indicator of anthropogenic pollution. Moreover, CO is a sink for the hydroxyl radical (OH), which in turn controls the levels of other important greenhouse gases, like methane [4,5]. Finally, the analysis of the CO-ozone correlation, which is negative in the stratosphere and dependent on season and latitude in the upper troposhere, provides useful information to distinguish between the two main tropospheric ozone sources, chemistry and transport [6,7].

For all these reasons, CO has been measured in numerous campaigns with different platforms [8,9,10,11,12,13,14] and taken using a large number of different measurement techniques. A review of all these methods, with particular attention to quite recent high sensitivity techniques, such as Cavity Ring Down Spectroscopy (CRDS), Cavity-Enhanced Absorption Spectroscopy (CEAS) and Integrated Cavity Output Spectroscopy (ICOS), often in combination with Quantum Cascade Lasers (QCL), can be found in [15].

Our goal is to realize a portable and versatile spectrometer for in situ CO measurements in the troposphere and in the stratosphere, that can be easily installed onboard both stratospheric aircraft or balloons and able to operate completely unattended for long periods. In particular such a spectrometer must fulfill the following requirements: (1) it must feature small size, light weight and low power consumption, to be versatile enough to be installed on different platforms with only few mechanical adjustments, and without demanding requirements for space and power; (2) it must be robust enough to guarantee proper operation when exposed to strong mechanical vibrations and shocks, and when working in harsh environment (with pressure variable between 10 and 1000 mbar, and external temperature variable in the range −80 ${}^{\circ }$C to +40 ${}^{\circ }$C); (3) it must be able to work unattended, without the presence of an operator, not only during the flight, but during the whole measurement campaign; (4) it must have a resolution time of about 1 s, in order to have a spatial resolution of about 200 m when used on board aircraft; (5) it must provide a large dynamic range for CO mixing ratio, from 200 down to a few ppbV, in order to provide measurements in both troposphere and stratosphere in tropical and arctic regions; (6) it must guarantee a sensitivity, meant as the minimum detectable mixing ratio variation, of about 1–2 ppbV, in order to register even small concentration changes due to air transport, and to allow a comparison between experimental data and atmospheric transport models.

In this paper we report the design, realization, and laboratory and in-field operation of a new instrument which is a good compromise between compactness and versatility on one side and performance on the other side, and which fulfills all the 6 previous requirements. The instrument was tested during a measurement campaign in summer 2017 in Nepal, on board the stratospheric aircraft Miyashichev M55 Geophysica, able to operate at an altitude up to 21–22 km, where it replaced the old CO sensor COLD (Cryogenically Operated Laser Diode) [14], which had operated during several campaigns since 2005.

With respect to the previous analyzer, the new device COLD2 (Carbon Oxide Laser Detector 2) maintains mainly the same performance (5) and (6), but with an increase of the resolution time of a factor of 4, and provides great advantages with respect to points (1) and (3). In particular, the old sensor had a total weight of 85 kg and was formed by 4 pressurized, separated boxes (optics, computer, laser driver, digitizer), with the one containing the optical setup large enough (55 × 42 × 42 cm${}^{3}$) to house a liquid nitrogen Dewar, for a cryogenic lead salt laser. This source required a pressurization system to keep the boiling temperature of liquid nitrogen constant, and the presence of an operator for the refill of the liquid nitrogen. Several technological improvements were adopted for the realization of the new sensor, aiming in particular to the reduction of size and weight, and to the capability of operation without any service by an external operator. Moreover, a new multipass cell, equipped with a piezo actuated mirror, has been exploited.

## 2. Materials and Methods

### 2.1. Selection of the Technique and of the CO Absorption Line

Several optical techniques can fulfill, and also exceed, the performance requirements (4), (5) and (6) described in the previous Section [15]. However other features must be taken into account, in order to reach the goal of both portability and completely unattended operation in harsh environments for long periods.

In order to limit the maintenance of the sensor to rare and short periods between different campaigns, the selected technique must guarantee absolute measurements and good reliability without on-the-ground test or calibration procedures made by trained personnel. For high-altitude airborne or balloon-borne devices exploiting non-absolute techniques, the large variation of the measurements conditions, in terms of change of pressure and temperature, forces either an automatic in-flight calibration for all working conditions, or the presence of a pressurization and temperature-stabilization system, which maintains the sample in the same conditions of the on-ground calibration during the flight. In both cases, size and weight of the instrument would suffer a substantial increase, coupled to a more complex mechanical design, and the necessity of certified calibration standards. As a consequence, we preferred not to adopt techniques which require calibration procedures, as Wavelength Modulation Spectroscopy (WMS), CEAS or ICOS , even if they guarantee higher sensitivity and lower noise.

Moreover, the sensor must operate for long periods on board atmospheric platforms in a harsh environment, and can suffer the problem of humidity and moisture. During descents, with increasing pressure, an unpressurized device is filled with air, which is warmer than the inside of the box. If the water vapor content in the air is high enough (and in tropical regions this is very frequent), condensation can occur on the cold surfaces of the device, with a reduction of the mirror reflectivity, and risk of short-circuit for electrical components. In addition, due to vibrations, shocks and large temperature gradients, a slight laser beam misalignment could arise with time. CRDS technique, whose performance is highly affected by degradation of the surface quality of the mirrors and by beam misalignment, can suffer these kind of problems.

We need a calibration-free optical technique which can provide absolute concentration values, and which is not strongly affected by degradation and misalignment. A simple approach that can fulfill all these demanding requirements is the well-known direct Tunable Diode Laser Spectroscopy (dTDLS), in which the transmission spectrum of a single-mode diode laser is recorded by scanning its emission wavelength across a molecular absorption line, and the concentration is directly inferred by this transmission signal, according to the Beer-Lambert law. The dTDLS technique, in spite of a reduction of sensitivity with respect to other low noise methods, has the great advantage to be a calibration-free technique [16] which provides the absolute mixing ratio value by using some molecular parameters (in particular the molecular linestrength) listed in molecular databases (we adopted HITRAN [17]). As a consequence, the accuracy of the absolute concentration value depends on the uncertainty of the HITRAN parameters and it is lower with respect to methods which make use of calibration procedures. However, most of the mid-infrared CO HITRAN line strengths are known with an accuracy better than 1% [18]. For typical CO mixing ratio in the stratosphere (tens of ppbV or less in the polar regions), the contribution of the linestrength uncertainty to the accuracy of the absolute value becomes less than 1 ppbV, which is better than the required sensitivity.

Another great advantage of dTDLS is that the transmission signals are always normalized with respect to the instantaneous laser power, so that the measurements are not affected by the power reduction due to mirror degradation or slight misalignment (apart from a reduction of the precision due to a deterioration of the signal-to-noise ratio).

For all these reasons, we have decided to use the dTDLS approach (as already used in the former version of the instrument [14]). The new device COLD2 is based on dTDLS in combination with a multipass cell and a room temperature QCL (L12004-2190H-C, Hamamatsu Photonics K.K., Hamamatsu City, Japan), emitting in single mode around 2190 cm${}^{-1}$, where CO shows strong rovibrational absorptions. The use of a QCL, which allows a room temperature operation, means a great improvement in terms of both size and simplicity with respect to a cryogenic sensor. Moreover the small intrinsic linewidth of a QCL (tipically few hundreds of Hz [19]) makes this source particularly suitable to be used in atmospheric applications, where the laser frequency must scan across molecular absorptions with linewidths that in the stratosphere can reduce down to few hundreds of MHz.

**Figure 1:**
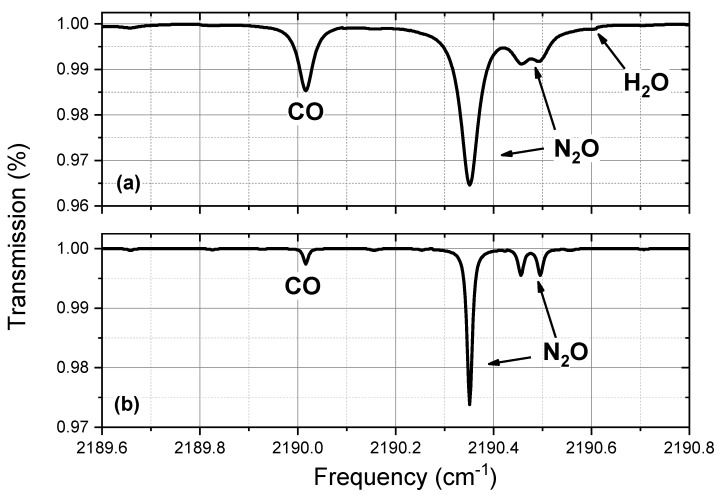
HITRAN simulation of the spectral region around the selected CO line. (**a**) Simulation of the tropospheric spectrum at 300 mbar (about 10 km altitude). The CO mixing ratio is 100 ppbV, the N${}_{2}$O mixing ratio is 325 ppbV and we overestimated the water vapour mixing ratio to 1000 ppmV. (**b**) Simulation of the stratospheric spectrum at 80 mbar (about 20 km altitude). The CO mixing ratio is 20 ppbV, the N${}_{2}$O mixing ratio is 260 ppbV and the water vapour (not visible) mixing ratio is 10 ppmV.

Another important point is the choice of the CO absorption line under analysis. Compatibly with the available laser sources, the selected spectral region must be a good compromise between strong absorption and selectivity. In particular, the selected CO absorption line at 2190.02 cm${}^{-1}$ (with an HITRAN linestrength of 3 × 10${}^{-19}$ cm/molecule), has been identified in such a way to avoid any kind of interference with other molecular absorptions, as water vapor and nitrous oxide (N${}_{2}$O), whose concentrations can change significantly in the different regions of the atmosphere. In an interval of 1 cm${}^{-1}$ around the selected CO line, the only H${}_{2}$O absorption, with an HITRAN linestrength larger than 10${}^{-24}$ cm/molecule, is 0.6 cm${}^{-1}$ far from the CO. For a pressure of 0.7 atm (at approximately 3 km above ground), the CO full width at half maximum (FWHM) is about 0.07 cm${}^{-1}$ and it becomes lower with increasing altitude. It means that in the worst case H${}_{2}$O and CO are far each other about 8.6 times the CO FWHM, and we can conclude that in this spectral region no interference between CO and water vapor is present. With respect to N${}_{2}$O, the interference condition is only slightly worse, because N${}_{2}$O and CO are separated of 0.33 cm${}^{-1}$, that is about 5 times the CO FWHM, in the worst case of few km above ground. The HITRAN simulated spectra for CO, N${}_{2}$O and H${}_{2}$O, around the selected CO line, in typical tropospheric and stratospheric conditions, are shown in Figure 1.

### 2.2. Opto-Mechanics

**Figure 2:**
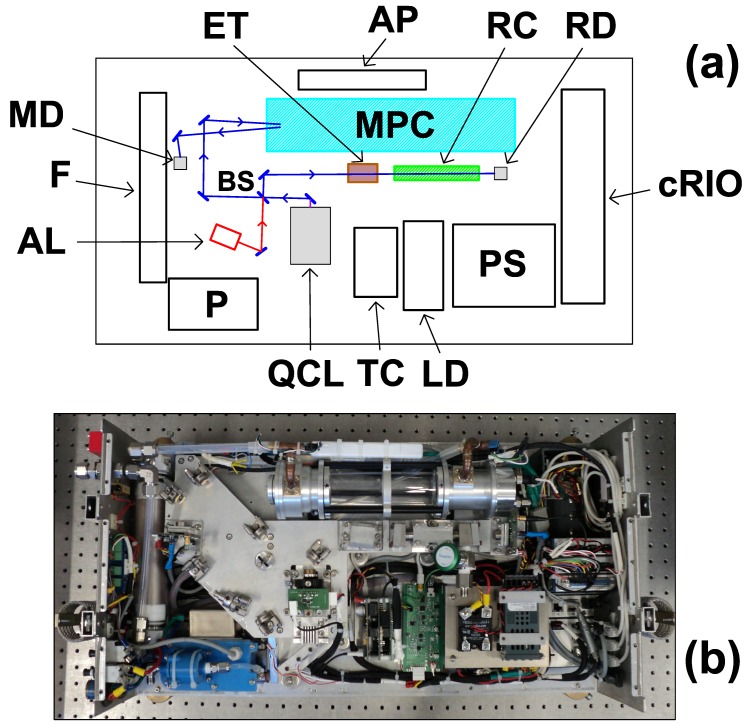
(**a**) Sketch and (**b**) picture of COLD2 instrument. QCL: Quantum Cascade Laser; BS: beam-splitter; ET: etalon; RC: reference cell; RD: reference detector; MPC: multipass cell; MD: main detector; AL: red laser for alignment; F: humidity filter; LD: QCL driver; TC: QCL temperature controller; cRIO: CompactRIO crate; PS: power supply; P: pump; AP: access point for wireless remote control.

The optical line of COLD2 is described in Figure 2. Once collimated by an aspheric lens (C036TME-E, Thorlabs, Newton, NJ, USA, effective focal length 4 mm, AR coated 3–5 μm), the laser beam is split by a 50:50 beam splitter (BSW505, Thorlabs, Newton, NJ, USA). Half of the beam is reflected towards a home made ZnSe etalon with a Free Spectral Range (FSR) of 0.071 cm${}^{-1}$ and a 10 cm reference cell, filled with pure CO (pressure < 1 mBar). The reference cell and the etalon are used for checking the stability of the laser emission frequency and for relative frequency calibration, respectively. The transmitted beam crosses a multipass, astigmatic Herriott cell (AMAC-36LW, Aerodyne, Billerica, MA, USA), and then hits a detector. This cell features (36.38 ± 0.04) m optical path with 194 passes. A peculiar characteristic of this cell is that the rear mirror is mounted on a piezo actuator (CEB-20D64, CUI Inc., Tualatin, OR, USA), which can be used to vibrate the mirror along the optical axis. The effect of this feature will be shown in Section 3.1.

The laser source is a QCL (L12004-2190H-C, Hamamatsu Photonics K.K., Hamamatsu City, Japan), emitting about 25 mW at 2190 cm${}^{-1}$, at the operating current of 860 mA and the temperature of 27 ${}^{\circ }$C. The detectors are two InAsSb photovoltaic detectors (P11120-201, Hamamatsu Photonics K.K., Hamamatsu City, Japan, D${}^{*}$ = 4.9 × 10${}^{9}$ cm·Hz${}^{1/2}$/W), which are temperature stabilized by Peltier elements. The use of non cryogenic source and detectors, in combination with a selection of both commercial and home made electronics which do not require air cooling or fans, make the sensor able to operate without pressurization. As a consequence, size and weight can be strongly reduced, and the sensor is entirely contained in a single compact, unpressurized box of size 80 × 25 × 41 cm${}^{3}$.

**Figure 3:**
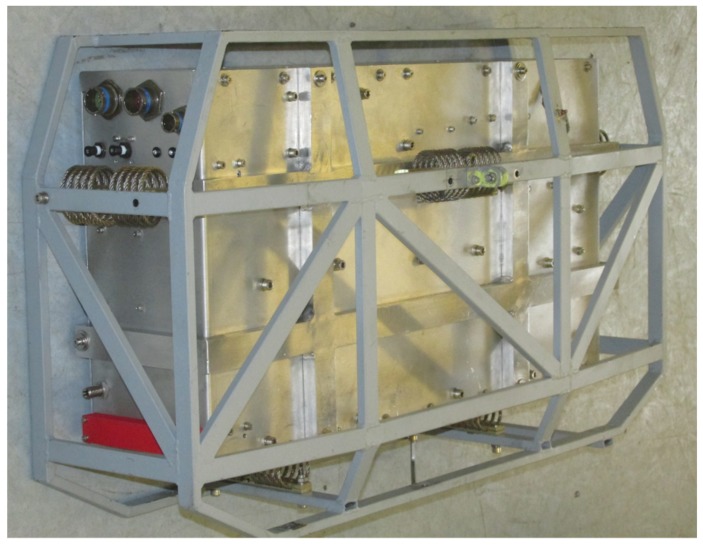
Picture of the external appearance of COLD2 inserted in the rack, before installation into the dome of the M55 Geophysica stratospheric aircraft.

The anti-vibration mechanism and the system for external air sampling have been designed for installation in the dome of the M55 stratospheric aircraft, but they can be easily adapted to other platforms. The box is connected to a frame, which is mounted into the M55 aircraft, via 5 spring dampers (CAVOFLEX H40-127-42-50-A2, Vibrostop, Milano, Italy), which have been chosen and tested in order to face 12 g vibrations and shocks [20]. The optical breadboard is attached to the interior of the box by 11 bolts, with rubber dampers, in order to decouple the movements of the box from the optics. A picture of the external appearance of COLD2, when inserted in the frame, is shown in Figure 3, where also four of the five dampers are visible. The instrument is robust and compact and features a total weight of 23 kg (including dampers).

The air flow in the multipass cell is provided by a probe, placed outside the aircraft on the top of the dome, behind the cockpit. The probe is connected by flexible Teflon tubing (with internal diameter of 8 mm) to the instrument. The air entering the instrument crosses a home made drying filter, based on silica gel, then goes through the multipass cell. Since the probe does not provide any dynamic pressure, a pump (G 24/07-N, Gardner Denver Thomas GmbH, Munich, Germany) is used to obtain a flow of about 18 L/min, so that the 0.3 L volume of the multipass cell is flushed once per second. Two Silicon Ceramic, temperature compensated piezoresistive pressure sensors (SSC series, Honeywell, Golden Valley, MN, USA), located at the entrance and exit of the multipass cell, provide the air pressure and temperature values, simultaneously to the detection of the transmission spectrum, with an accuracy of ±2%.

As described in Section 2.1, an unpressurized box can suffer a humidity problem during the descent of the aircraft, due to water condensation on the cold surfaces of the analyzer. To solve the problem and to reduce the degradation of the surface quality of the mirrors after several flights, the exhaust of the pump is released inside the analyzer, so that dry air purges the inside of the box continuously. Moreover, in order to remove any possible contamination by CO along the optical path inside the instrument, we added a filter to the pump exhaust, filled with Carulite${}^{®}$ 300. This is a catalyst, consisting of manganese dioxide/copper oxide which, in presence of oxygen, converts CO to CO${}_{2}$.

### 2.3. Electronics

**Figure 4:**
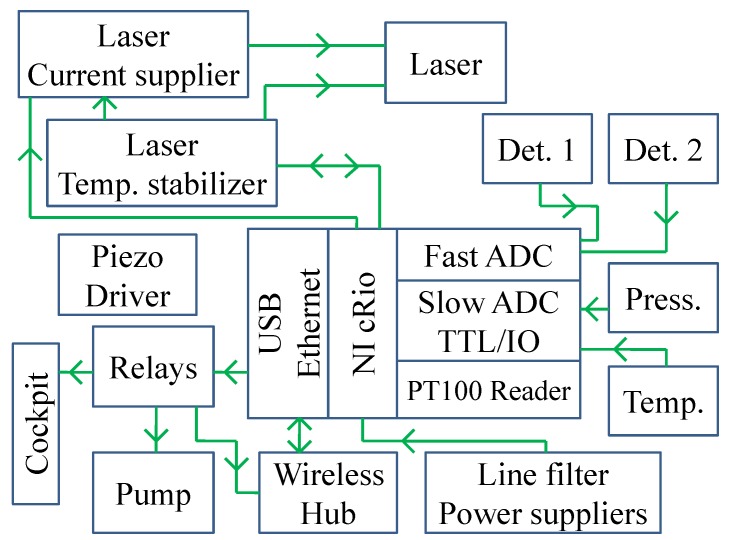
Block diagram of the electronics.

All the electronics are positioned inside the analyzer as shown in Figure 2. A block diagram of the electronics is shown in Figure 4. The core of the instrument is a CompactRIO crate (cRIO 9068, National Instruments, Austin, TX, USA), which combines a dual-core processor, a reconfigurable field programmable gate array (FPGA), and 8 slots for plug-ins with 3 modules inserted: a DAQ module for fast acquisition of the main and reference signals (4 independent channels, simultaneous acquisition rate at 1 MHz, resolution 16 bits (NI9223)); a 32-Channel TTL digital Input/Output module for system control (NI9403); and 4-Channel Module at 24-Bit for thermocouple reading for temperature housekeeping (NI9217). As a matter of fact, smaller versions of cRIO are available (four slots), but they feature a lower performances computer and, above all, a narrower operational temperature range.

The QCL Current Driver and Temperature Controller are home-made and provide a flat current noise spectrum of 1 nA/Hz${}^{1/2}$ in the range 10 Hz–1 MHz [19], and a temperature stability better than 1 mK. They are connected to the cRIO via the USB bus, and are equipped with microprocessors, which allow a complete control of the laser parameters. They provide the startup and the shutdown sequences, even if the connection with the cRIO is lost.

The last component of the electronics is the driver for the piezo actuator of the multipass cell.

Software is tightly linked to the electronics architecture. The whole system is controlled by a home made LabVIEW program. The cRIO computer can be managed via a web interface (either wired, or wireless connection), which shows all the parameters of the instrument, namely the laser operating conditions, the waveforms acquired by the two detectors, temperatures and pressure, and provides a rough estimation of the CO mixing ratio. Several safety checks are implemented in both hardware and software. In particular, great care has been taken of the protection of the laser, as QCL’s are much more affected by electric disturbances than near infrared Distributed FeedBack lasers are. For instance, no reverse bias is allowed. The laser is always switched on and off gradually, following a preset current ramp. Anyway, even in the case of a lack of power during flight, a capacitor driven circuitry allows a smooth decay of the laser current, without any spike.

A pressure sensor, located outside the multipass cell, is used to switch on (and off) the pump only at heights above (below) 3500 m. This is to avoid sucking particulate in the lower atmosphere. Moreover, the QCL is turned on immediately after the pump during ascent, and turned off immediately before the pump during descent. This is for two reasons: to avoid any disturb to the laser due to the current transients at pump start/stop, and because after landing the power to all instruments is abruptly interrupted.

The total power requirement for the normal operation of the spectrometer is about 85 W at 28 V DC, mainly due to the laser driver and temperature controller necessary for the normal operation of a QCL. The internal power supply consists of two DC/DC converter (Traco Power) with an output of ±24 V DC with 7.5 A. An Eurotherm 2132 controller maintains the optical breadboard at a temperature around 25 ${}^{\circ }$C by using two silicon rubber heaters, supplied by the 115 V, 400 Hz power line of the aircraft.

The spectrometer can work completely unattended and, if the case, can be remotely controlled via WiFi. During the first measurement campaign of COLD2, we used this option on the apron, before take-off, to check that everything was properly working, and then the Wi-Fi router was disabled just before take-off.

### 2.4. Acquisition and Data Processing

The CO mixing ratio value is inferred by the transmission signal registered by the main detector, according to the Beer-Lambert law. The QCL emission frequency is scanned across the selected CO absorption at 2190.02 cm${}^{-1}$, by modulating the laser current with a ramp signal. The laser tunability allows a frequency scan of about 1 cm${}^{-1}$. The modulation signal has a repetition frequency of 1 kHz and consists of 2 parts: a linear ramp with a duration of 900 μs and a region of 100 μs during which the laser is turned off to get the detector dark signal.

**Figure 5:**
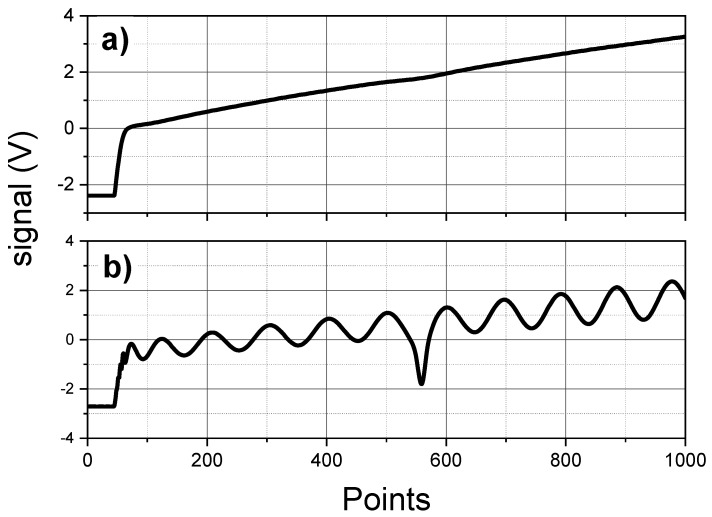
Example of a typical acquired signals. (**a**) main channel with ambient CO; (**b**) reference channel with reference cell containing CO at low pressure and etalon.

The reference and main signals are synchronously acquired at 1 MSample/s with a resolution of 16 bits. Each 1000-points signal is averaged by the FPGA and saved for a post-processing. An example of acquired spectra is shown in Figure 5. Also pressure and temperature are saved together with each acquisition. The recorded data (averaged spectra, time, pressure, temperature and housekeeping) are stored on a usb flash drive. For a typical integration time of 1 s (corresponding to 1000 averages), the rate of acquired data is about 60 MB/h.

The averaged spectra are analyzed with a post-processing software. The main signal, after subtracting the detector background at zero-power (shown in the beginning part of the main signal of Figure 5), is fitted with the exponential of a Voigt profile multiplied by a two-order polynomial, which simulates the sloping power due to the ramping of the driving current. The four-Lorentz Puerta-Martin approximation [21,22] was used as Voigt profile. The only fitted parameters are the amplitude and the center frequency, while the Lorentzian and Gaussian FWHM are kept fixed and calculated as a function of temperature, pressure and molecular parameters according to the HITRAN database [17]. In this approach, the frequency calibration of the x-axis becomes essential. Consequently, the corresponding reference signal is fitted according to the etalon transmission equation, multiplied by a two-order polynomial and by the absorption signal in the reference cell. The frequency scale for each acquisition can be determined by the fitted parameters and the etalon FSR.

The presence of undesired optical fringes in the main signal, due to spurious etalon effect between optical surfaces, usually affecting spectroscopic measurements, is taken into account by inserting in the fitting function one or more sinusoidal curves.

The CO concentration *N* is calculated as a function of the CO integrated absorbance (calculated according to the fitting parameters of the Voigt function), the path length (listed in the datasheet of the multipass cell), and the CO line-strength (listed in the HITRAN database). Finally, the mixing ratio MR is obtained according to the Equation:

(1) $$MR=\frac{N}{N_{0}}\frac{T}{T_{0}}\frac{P_{0}}{P}$$

where *N* is the calculated molecules concentration in cm${}^{-3}$, *P* and *T* are the measured values of pressure (in atm) and temperature (in K), $P_{0}$ = 1 atm, $T_{0}$ = 296 K and $N_{0}$ is the Loschmidt number ($N_{0}$ = 2.470×10${}^{19}$ cm${}^{-3}$).

The mixing ratio uncertainty is mainly determined by the following contributions: the accuracy of the temperature and pressure measurement (about 2% respectively), the accuracy of the CO line strength according to the HITRAN database (1%) and the uncertainty in the fitting procedure (about 1%). The resulting total accuracy for CO mixing ratio values, given by the square root of the quadrature sum of the single contributions, is 3%. We neglect the uncertainty on the cell pathlength, as for an astigmatic Herriott cell, the error affecting this value is due to the uncertainty in the curvature radii of the mirrors, which is typically in the range 0.1–0.2% [23], a value much lower than the other described contributions.

A detailed description of the data processing and of the formalism used to calculate the accuracy can be found in [14].

## 3. Results

### 3.1. Laboratory Tests and Performances

One of the main features which limit the performances of sensors based on multipass cells is the presence of optical fringes, due to undesired beam superpositions inside the cell, which cause a spurious etalon effect. Even if a post-processing analysis can reduce this effect, the best solution should be to cancel the optical fringes before the acquisition. For this goal, COLD2 is equipped with a multipass cell whose rear mirror can vibrate along the optical axis by means of a piezoelectric transducer (PZT). The mirror vibration during the acquisition time results in a random phase averaging, and a consequent reduction, of the optical fringes. In principle the straightforward voltage supply for the PZT is a sinusoid at a fixed frequency, coincident with the resonance of the system PZT plus mirror, which is around 350 Hz. However we found that this resonance frequency depends on temperature and pressure, and the resonance peak is quite narrow (∼10 Hz). In order to obtain a good fringes reduction for a large range of pressure and temperature, we decided a different approach. A random voltage level, in the interval 0–16.2 V, is generated and applied to the PZT every 650 μs.

**Figure 6:**
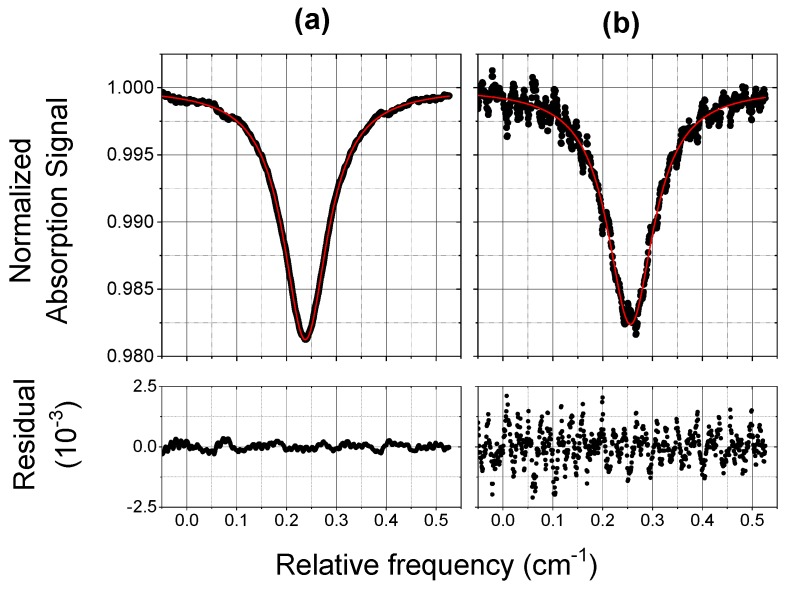
Normalized absorption spectra of a 125 ppbV CO mixture (scatter) and Voigt fit results (red line). The residual of the fitting procedure is shown in the bottom graph. The acquisition time is 1 s. The measurement was carried out at ambient pressure and at a temperature of 295 K. The effect of the PZT in the multipass cell is also shown: (**a**) PZT on; (**b**) PZT off.

**Figure 7:**
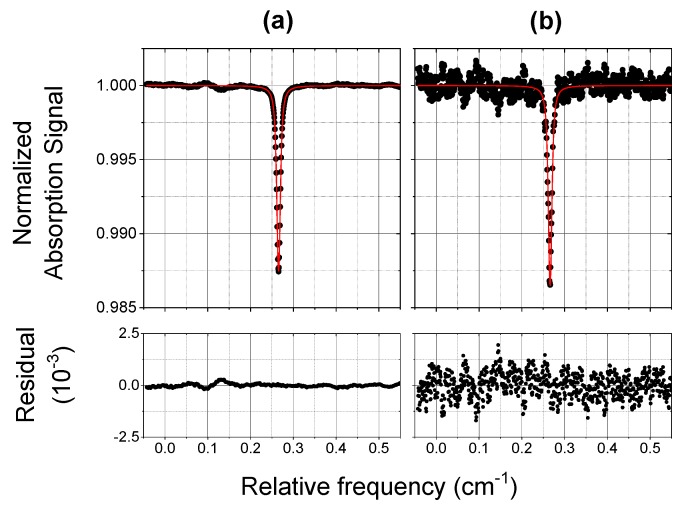
Normalized absorption spectra of a 125 ppbV CO mixture (scatter) and Voigt fit results (red line). The residual of the fitting procedure is shown in the bottom graph. The acquisition time is 1 s. The measurement was carried out at a pressure of 70 mbar and at a temperature of 295 K. The effect of the PZT in the multipass cell is also shown: (**a**) PZT on; (**b**) PZT off.

In order to test the PZT operation and to verify the reliability of the inferred concentration values, we used a commercial CO mixture in synthetic air, with a mixing ratio of 125 ppbV, known with a standard uncertainty of 15 ppbV. Figure 6 and Figure 7 show typical normalized absorption spectra of the CO mixture detected by COLD2 in 1 s (with 1000 averages), at two different pressures of 970 mbar and 70 mbar. Plots (a) are the absorption profiles registered with PZT on and plots (b) are the corresponding profiles with PZT off. The results of the Voigt fitting procedures and the corresponding residuals are also reported. From the spectra of Figure 6 and Figure 7 we retrieve a CO mixing ratio respectively of (125 ± 4) ppbV and (122 ± 4) ppbV, where we have considered an accuracy of 3%. The obtained results are in perfect agreement with the concentration of the used mixture.

We assume as signal-to-noise ratio S/N, the ratio between the normalized absorption signal and the conservative value of two times the standard deviation (2σ) of the Voigt fit residual, and we estimate the COLD2 sensitivity as the mixing ratio for which S/N = 1. As for the spectra shown in Figure 6, the S/N is about 70 (corresponding to a sensitivity of about 2 ppbV) with piezo on and about 10 (corresponding to a sensitivity of about 12 ppbV) with piezo off. As for the spectra shown in Figure 7, the S/N is about 80 (corresponding to a sensitivity of about 1.5 ppbV) with PZT on and about 10 (corresponding to a sensitivity of about 12 ppbV) with PZT off . We can conclude that the effect of the PZT increases the sensitivity of a factor between 7 and 8.

In order to evaluate the long-term stability of the instrument, the CO spectra of the mixture (at the two different pressures of 970 mbar and 70 mbar) were acquired for 1 h at the rate of 1 Hz (1000 averages). By assuming as COLD stability the standard deviation of the mixing ratio values, we obtain that at ambient pressure the mean value of the CO mixing ratio is 118 ppbV with a standard deviation of 9 ppbV and at lower pressure the mean value of the CO mixing ratio is 116 ppbV with a standard deviation of 5 ppbV. We can conclude that the 1-hour stability of the instrument, at an integration time of 1 s, is about 7.5% at room pressure and it improves to 4% at a pressure of 70 mbar.

**Figure 8:**
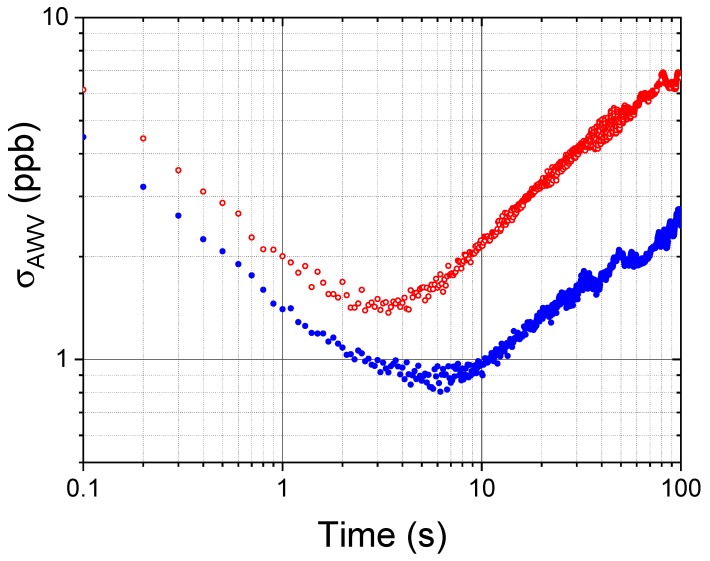
Allan-Werle Variance ($\sigma_{AVW}$) plot of the CO mixing ratio as a function of the integration time, for 30 min measurement of a CO mixture (mixing ratio 125 ppbV) at a rate of 10 Hz, at a temperature of 295 K and for two different pressures: 980 mbar (red open circles) and 50 mbar (blue closed circles)

In order to find the optimum averaging time of COLD2 and to evaluate the ultimate sensitivity of the instrument, we carried out an Allan-Werle variance analysis [24]. In this case, the CO spectra of the mixture (at the two different pressures of 980 mbar and 50 mbar) were acquired for 30 min at the rate of 10 Hz (100 averages). The obtained results are reported in Figure 8, where the optimum averaging time is around 3 s at room pressure and around 6 s at low pressures. If the acquired spectra are averaged for longer times, the frequency-dependent drifts (mainly due to changes in the background spectra during averaging) become predominant and the averaging procedure is not useful anymore. We can conclude that the COLD2 sensitivity in standard conditions of operation (1 s of acquisition time) is 1.5–2 ppbV, depending on pressure, as already estimated from the S/N analysis of the spectra in Figure 6 and Figure 7. In order to have a detection limit lower than 1 ppbV, the instrument should operate at a pressure lower than 1 atm with an acquisition time larger than 2 s. The best achievable sensitivity of about 800 pptV can be reached for about 6 s of integration time.

The reduction of the COLD2 performance, in terms of both stability and sensitivity, with the increase of the measurement pressure, is due to the fact that the absorption signal at room pressure is as broad as the laser scan and consequently it is difficult for the fitting protocol to clearly identify the background signal and the optical fringes. At lower pressure, when the absorption signals are narrower, the fitting procedure becomes more precise. Obviously we could increase the laser wavelength scan (with a larger amplitude of the current ramp), provided that the number of acquired samples correspondingly increases, in order to not reduce the spectral resolution and to clearly identify the absorption profile also at low pressures, when the molecular linewidths are narrower. Nevertheless with increasing samples per ramp, the sweep repetition frequency should decrease, with a corresponding increase of the acquisition time. As COLD2 has to operate at low pressures on board aircraft or balloons, we prefer not to increase the laser scan time, while maintaining the spectral resolution.

### 3.2. In-Field Performances

COLD2 was deployed onboard the M55 aircraft in the framework of the European Community FP7 Project StratoClim (Stratospheric and upper tropospheric processes for better climate predictions, www.stratoclim.org).

The instrument was installed in the dome of the aircraft and performed its first test flight from Kalamata (Greece) on 30 August 2016. Then, in July–August 2017 the main field campaign of StratoClim was carried out from Kathmandu (Nepal), with eight flights over Nepal, India and Bangladesh. COLD2 worked properly during all the flights with no failure and without any particular service by operators.

**Figure 9:**
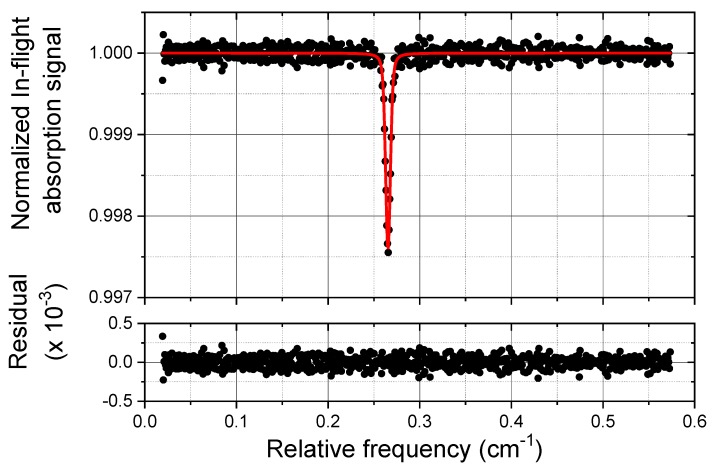
Example of a typical in-flight CO (36 ± 1 ppbV) normalized absorption spectrum (scatter) and Voigt fit results (red line). The residual of the fitting procedure is shown in the bottom graph. The acquisition time is 1 s. The measurement was carried out during the flight on the 29th July 2017 at an altitude of 19 km and at a temperature of 281.8 K.

A typical CO normalized spectrum, acquired during the flight on the 29th of July at a cell pressure of 20 mbar, is shown in Figure 9, together with the result of the Voigt fitting procedure and the corresponding residual. From this absorption profile, we retrieve a CO mixing ratio of (36 ± 1) ppbV, considering an accuracy of 3%, and a S/N of about 16 corresponding to a sensitivity of 2 ppbV. This in-flight sensitivity is only slightly worse with respect to the laboratory sensitivity of 1.5 ppbV obtained by the Allan-Werle variance analysis at integration time of 1 s and at low pressure (Figure 8), so that we can infer that the COLD2 performances are not significantly reduced during the in-flight operation.

**Figure 10:**
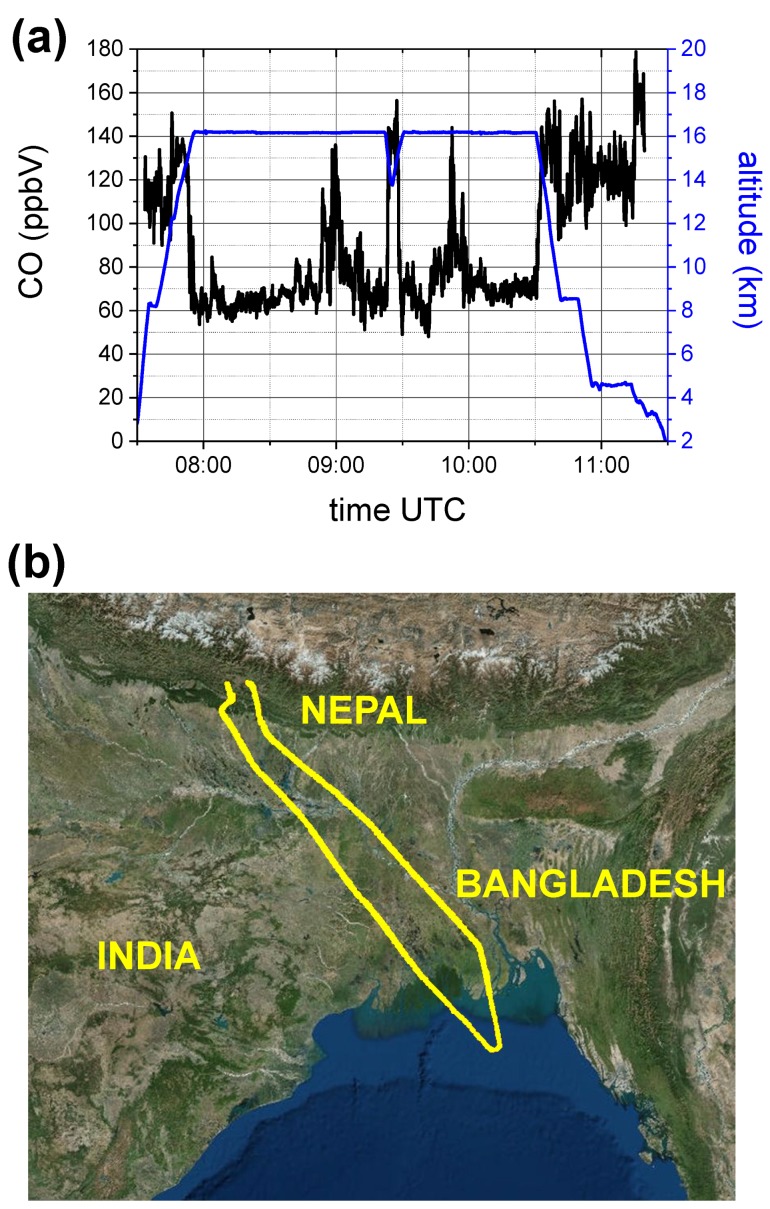
(**a**) COLD2 results on board the M55 stratospheric aircraft during the flight on the 6th August 2017 of the StratoClim campaign from Katmandu (Nepal). CO mixing ratio (black line) and aircraft altitude (blue line) vs UTC time. (**b**) map of the M55 route.

**Figure 11:**
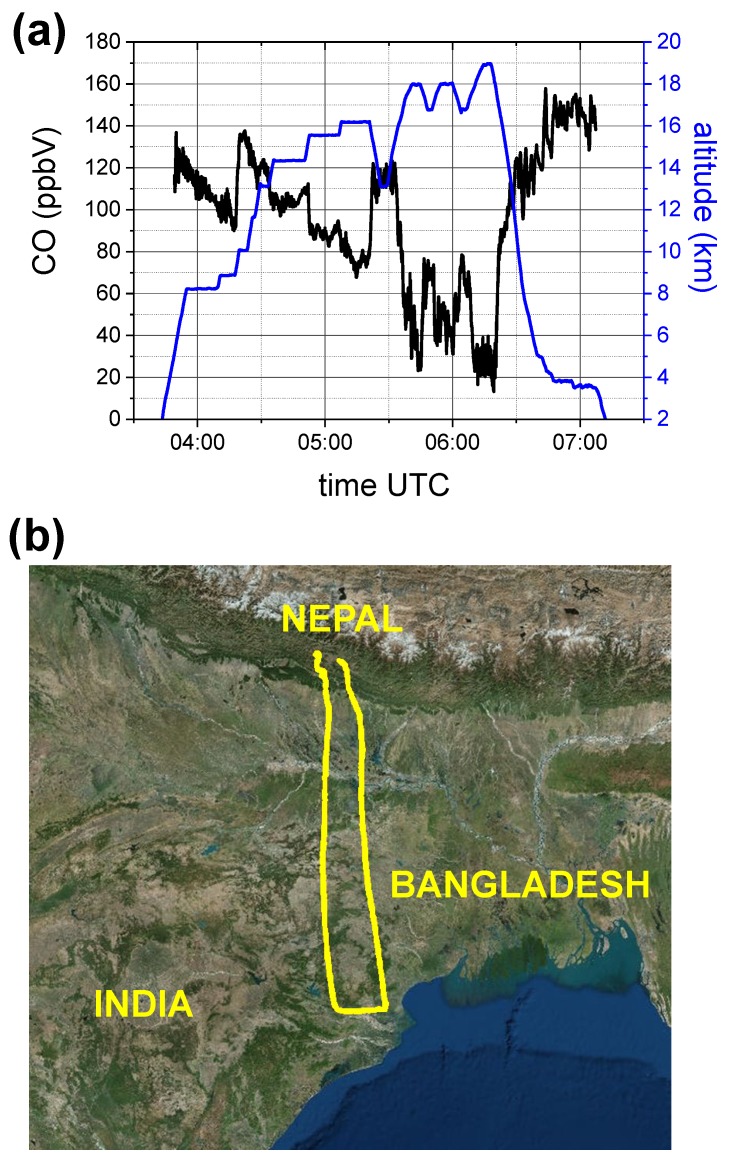
(**a**) COLD2 results on board the M55 stratospheric aircraft during the flight on the 8th August 2017 of the StratoClim campaign from Katmandu (Nepal). CO mixing ratio (black line) and aircraft altitude (blue line) vs UTC time. (**b**) map of the M55 route.

Two examples of CO time series, recorded during the flights on the 6th and 8th of August, respectively, are reported in Figure 10 and Figure 11, where also the aircraft altitude is shown. The CO mixing ratio decreases with altitude, reaching minimum levels of about 10–20 ppbV in the stratosphere, at a pressure of about 80 mbar and altitude around 19 km. While in Figure 11 the CO mixing ratio inversely follows the altitude profile, Figure 10 shows two CO enhanced layers at a constant altitude of about 16 km, respectively at UTC time 09:00 and 09:50. These two peaks are registered (with a time difference of 50 min) over the same region close to the Bay of Bengala, during the forth and back route of the aircraft. The specular profile of the two structures, with a double peak, clearly shows that the aircraft intercepted mainly the same intrusion of air, rich of CO, coming from the lower part of the troposphere due to convection activity.

**Figure 12:**
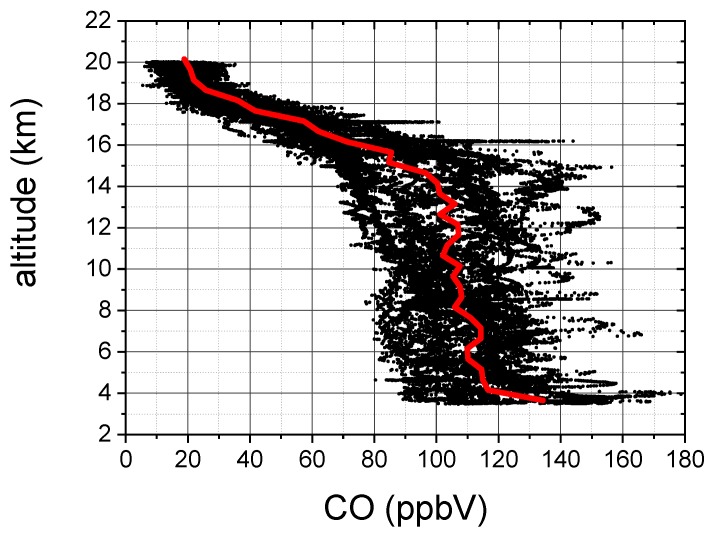
CO vertical profiles during the whole StratoClim campaign in Nepal in summer 2017 (black scatter). The red line is the CO mean vertical profile.

In Figure 12 we report the CO vertical profiles recorded during all flights of the campaign. The red curve is the CO mean vertical profile obtained with the following procedure: we calculated the mean value of the CO mixing ratio for altitude intervals of 500 m; then we discarded the CO values which differ more than 90% from the mean value (in order to skip the CO anomalies); and finally we re-calculated the mean value in the altitude intervals. The CO vertical profiles shown in Figure 12 are consistent with CO data measured by other aircraft or satellite instruments [8,9,25,26].

The analysis of the vertical profiles clearly shows that some CO positive anomalies (with respect to the CO mean value) can be observed in the upper troposphere. As an example, the CO enhanced layer shown in Figure 12 for altitudes around 16 km, corresponds to the two peaks of Figure 10.

The COLD2 CO data, and in particular the positive anomalies, must be analyzed jointly with other species measured by different instruments on board the M55 aircraft (i.e., aerosols, cloud, particles and ozone) and must be compared also with convective tracers from lagrangian models, to identify convective influence and source regions of potential pollutants in the upper troposphere. Moreover we have to carry out a comparison between COLD2 data and CO measured by both AMICA analyzer, the other in-situ CO instrument mounted on the M55 aircraft (*paper in preparation*) and by the remote-sensing Microwave Limb Sounder (MLS) [27].

All these studies are in progress and they will be the subjects of future papers.

## 4. Discussion

The set of laboratory and in-field results, described in Section 3, proved the COLD2 capability to fulfill all the 6 demanding requirements listed in Section 1. In particular:

(1) The selection of a calibration-free technique, as dTDLS, and the choice of optical and electronics components which work properly also without pressurization systems, allowed the realization of a portable device, entirely contained in one box only, that can be easily mounted on different platforms. The low weight, small size and low power consumption, with the only need of an access to sample the external air, make this instrument particularly suitable for installation on aircraft or balloons as piggyback in different missions.
(2) The robustness of COLD2 is guaranteed by a mechanical structure designed to damp vibrations and to decouple the movements of the box from the optical breadboard. Its robustness was demonstrated by its deployment onboard the M55 stratospheric aircraft, where it operated, without need of realignment and without any kind of damages, during 8 tropical flights in harsh environment, with pressure down to 50 mbar and external temperature variable between $40{\;}^{\circ }$C or higher (when the sensor was forced to stay for long periods inside the aircraft on the apron before take-off) and $-80{\;}^{\circ }$C (in the stratosphere).
(3) For all the period of the tropical campaign in Nepal, the sensor worked completely unattended without failure and without need of external maintenance, as calibration procedures. Although the sensor experienced high humidity during each aircraft descent, the signal-to-noise ratio did not suffer significant degradation and a procedure of mirror cleaning was not necessary.
(4) The COLD2 acquisition time for each 1000 points spectrum is 1 ms. The device time resolution depends on the number of averages and can be easily set to fulfill the requirements of different applications. An Allan-Werle analysis demonstrated that an integration time of 1 s allows a sensitivity of 2 ppbV at atmospheric pressure and that this sensitivity increases at lower pressure. COLD2 is able to work at 10 Hz with a sensitivity between 5 and 8 ppbV and can reach a sensitivity lower than 1 ppb for integration time of 6 s. For ballonborne operation, when the time resolution can be smaller with respect to an aircraft, COLD2 can reach sub-ppbV performance.
(5) The dTDLS technique in conjunction with a 16 bit acquisition system guarantees enough vertical resolution to register and analyze the CO absorption spectrum in a large range of pressure, temperature and concentration values. The COLD2 large dynamic range is demonstrated by the measurement of the CO vertical profiles during the campaign on board the M55 stratospheric aircraft (as shown in Figure 12) .
(6) The COLD2 sensitivity is not significantly reduced by in-flight operation and it was demonstrated to be around 2 ppbV @ 1 s of integration time.

Moreover this device can be used for the detection of other gases as well. For instance, by detuning the laser of only 0.3 cm${}^{-1}$ with respect to CO, it is possible to detect N${}_{2}$O. A larger laser scan can allow the simultaneous detection of CO and N${}_{2}$O.

## 5. Conclusions

We described design, operational features and performances of COLD2, a portable optical analyzer, specifically designed to be deployed for trace gas measurement onboard different stratospheric platforms for environmental and atmospheric research. The COLD2 external appearance is a small unpressurized box of size 80 × 25 × 41 cm${}^{3}$ and weight 23 kg (including the anti-vibration system). The normal operation power consumption is only 85 W. COLD2 was deployed onboard the M55 stratospheric aircraft for CO concentration measurements. The in-flight sensitivity of the instrument is around 2 ppbV @ 1 s of integration time, and the accuracy is 3%. COLD2 worked properly, completely unattended and without failure, during 8 scientific flights over Nepal, India and Bangladesh. No on-ground action by operators was required during the whole period of the measurement campaign.

For all these reasons, COLD2 is particularly suitable to be employed in future missions, also long lasting (even weeks), onboard stratospheric aircraft or balloons, for measuring CO or other trace gases. Although a lot of data can be acquired by satellites, in particular with limb instruments, their sparse temporal cover cannot ensure continuous records. A series of stratospheric flights to monitor CO, carried out with a light instrument, would be very useful to provide high temporal resolution series, impossible with the remote sensing instruments only, and to make a comparison with satellite measurements, which still present large discrepancies, especially in polar regions.

## Abbreviations

The following abbreviations are used in this manuscript:

CO	Carbon Monoxide
N${}_{2}$O	Nitrous Oxide
QCL	Quantum Cascade Laser
CRDS	Cavity Ring Down Spectroscopy
CEAS	Cavity-Enhanced Absorption Spectroscopy
ICOS	Integrated Cavity Output Spectroscopy
WMS	Wavelength Modulation Spectroscopy
dTDLS	Direct Tunable Diode Laser Spectroscopy
FWHM	Full Width at Half Maximum
FSR	Free Spectral Range
FPGA	Field Programmable Gate Array
PZT	piezoelectric transducer
